# Supplementary figures and images for: The MOV10 RNA helicase is a dosage-dependent host restriction factor for LINE1 retrotransposition in mice
Source: PLoS Genet. 2023 May 1;19(5):e1010566. doi: 10.1371/journal.pgen.1010566 (PMC10174503; doi:10.1371/journal.pgen.1010566)

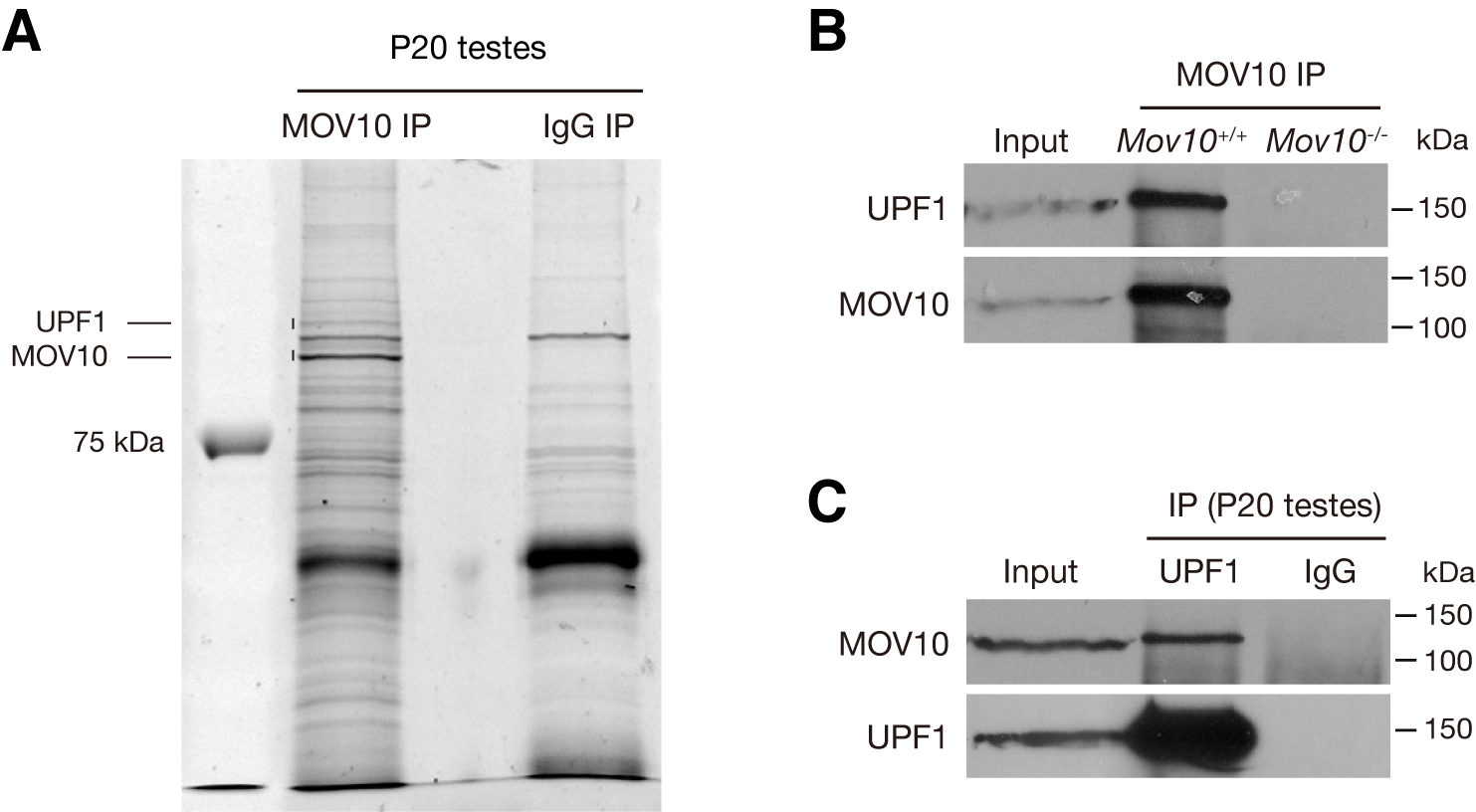

Supplement: S1 Fig — (A) Identification of MOV10-associated proteins in lysates from postnatal day 20 (P20) mouse testes by immunoprecipitation and mass spectrometry. The gel was stained with Coomassie Blue dye. The two bands indicated by vertical lines in the MOV10 immunoprecipitation lane were subjected to protein identification by mass spectrometry. (B) Co-immunoprecipitation analysis of MOV10 and UPF1 in P20 Mov10+/+ and Mov10-/- testes. IP was performed with anti-MOV10 antibody. (C) Reciprocal co-immunoprecipitation analysis of MOV10 and UPF1 in P20 wild type testes. IP was performed with anti-UPF1 antibody. (TIF) [file pgen.1010566.s001.tif]

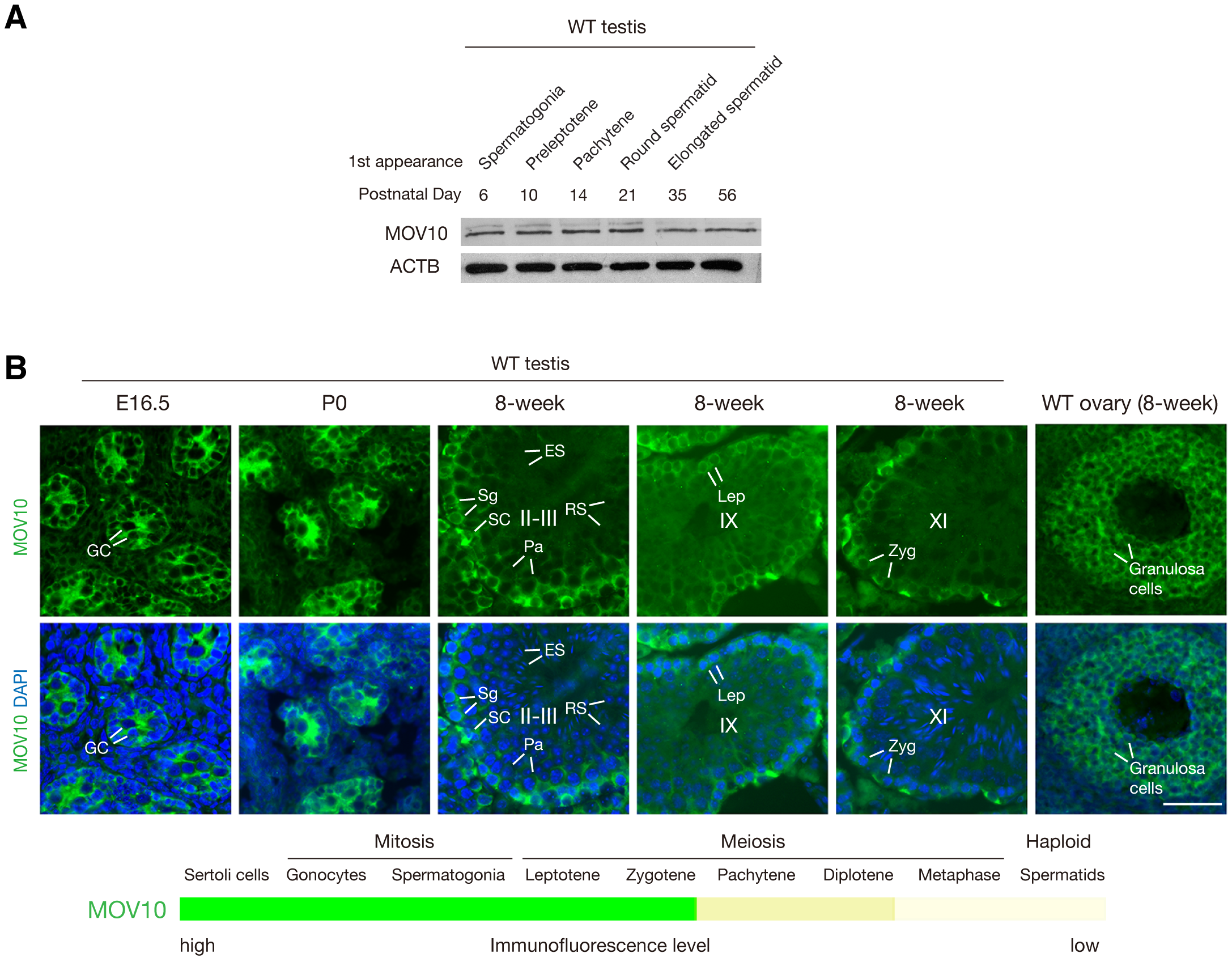

Supplement: S2 Fig — (A) Western blot analysis of MOV10 in developing mouse testes. The timing of the first appearance of spermatogonia, preleptotene spermatocytes, pachytene spermatocytes, round spermatids, and elongated spermatids in developing testes is shown. ACTB serves as a loading control. (B) Immunofluorescence of MOV10 in frozen sections of mouse testes at different ages and 8-week-old ovary. The expression levels of MOV10 are depicted in different colors at the bottom diagram. The stage of seminiferous tubules in 8-week-old testis is shown in roman numerals. GC, gonocytes; Sg, spermatogonia; SC, Sertoli cells; Lep, leptotene; Zyg, zygotene; Pa, pachytene; RS, round spermatids; ES, elongated spermatids. Scale bar, 50 μm. (TIF) [file pgen.1010566.s002.tif]

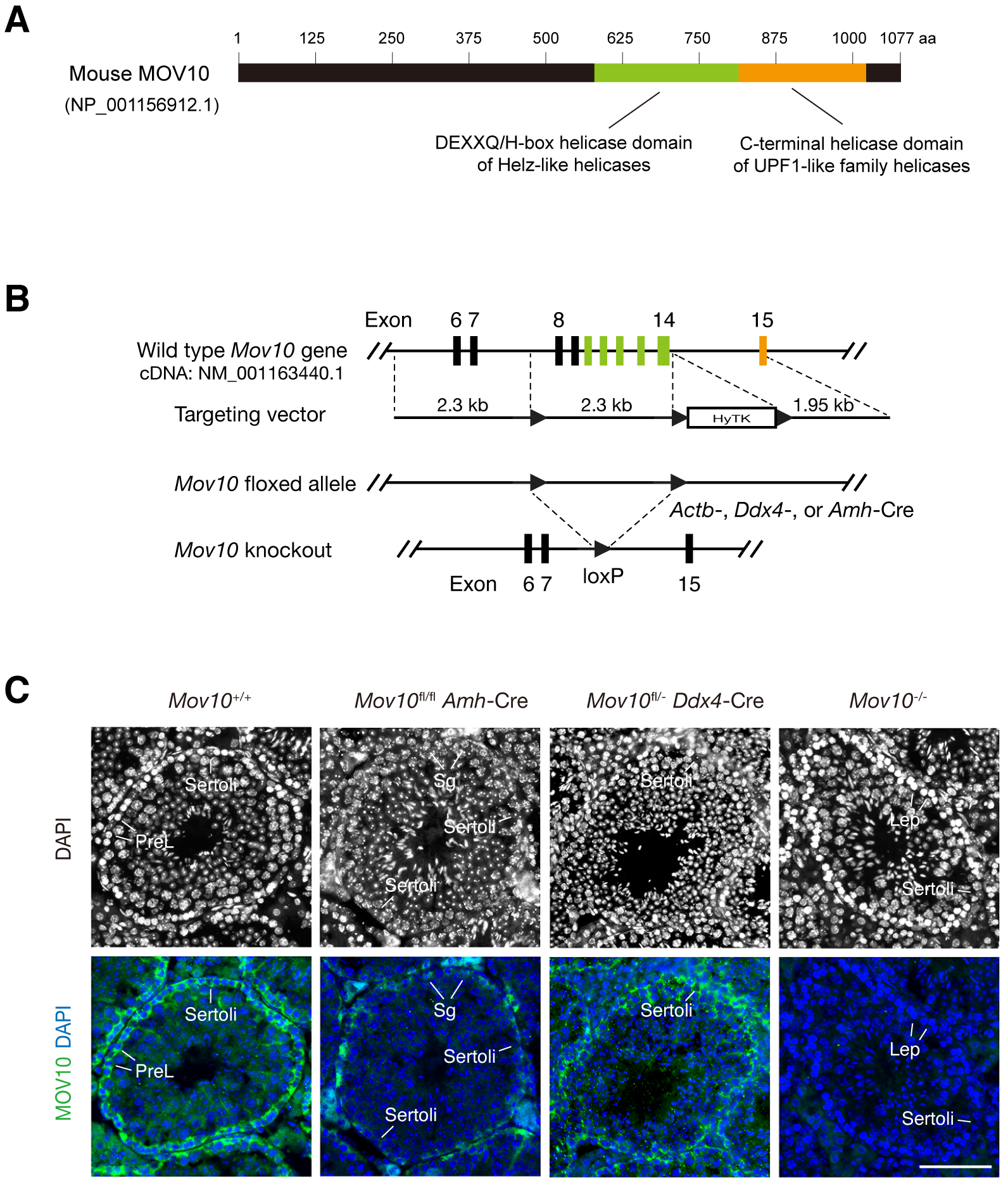

Supplement: S3 Fig — (A) Schematic diagram of the mouse MOV10 RNA helicase domains. The two different RNA helicase domains are color coded. (B) Targeted inactivation of the Mov10 gene. Targeting vector, floxed conditional allele, and knockout allele are shown. The Mov10 gene has 21 exons based on the cDNA under accession number NM_001163440.1. Deletion of exons 8–14 encoding aa 455–807 removes the first RNA helicase domain (in green) and results in a frame shift in the resulting transcript. The protein regions encoded by colored exons match those in panel A. (C) Immunofluorescence of MOV10 in frozen sections of testes from 8-week-old Mov10+/+, Mov10fl/fl Amh-Cre (Sertoli cell-specific conditional knockout), Mov10fl/- Ddx4-Cre (Germ cell-specific conditional knockout), and Mov10-/- (global knockout) mice. The meshwork-like green fluorescence pattern in Mov10fl/- Ddx4-Cre testis section is due to the expression of MOV10 in the cytoplasm of Sertoli cells, but is absent in Mov10fl/fl Amh-Cre testis section as expected. Sg, spermatogonia; PreL, preleptotene spermatocytes; Lep, leptotene spermatocyte; Sertoli, Sertoli cells. Scale bar, 50 μm. (TIF) [file pgen.1010566.s003.tif]

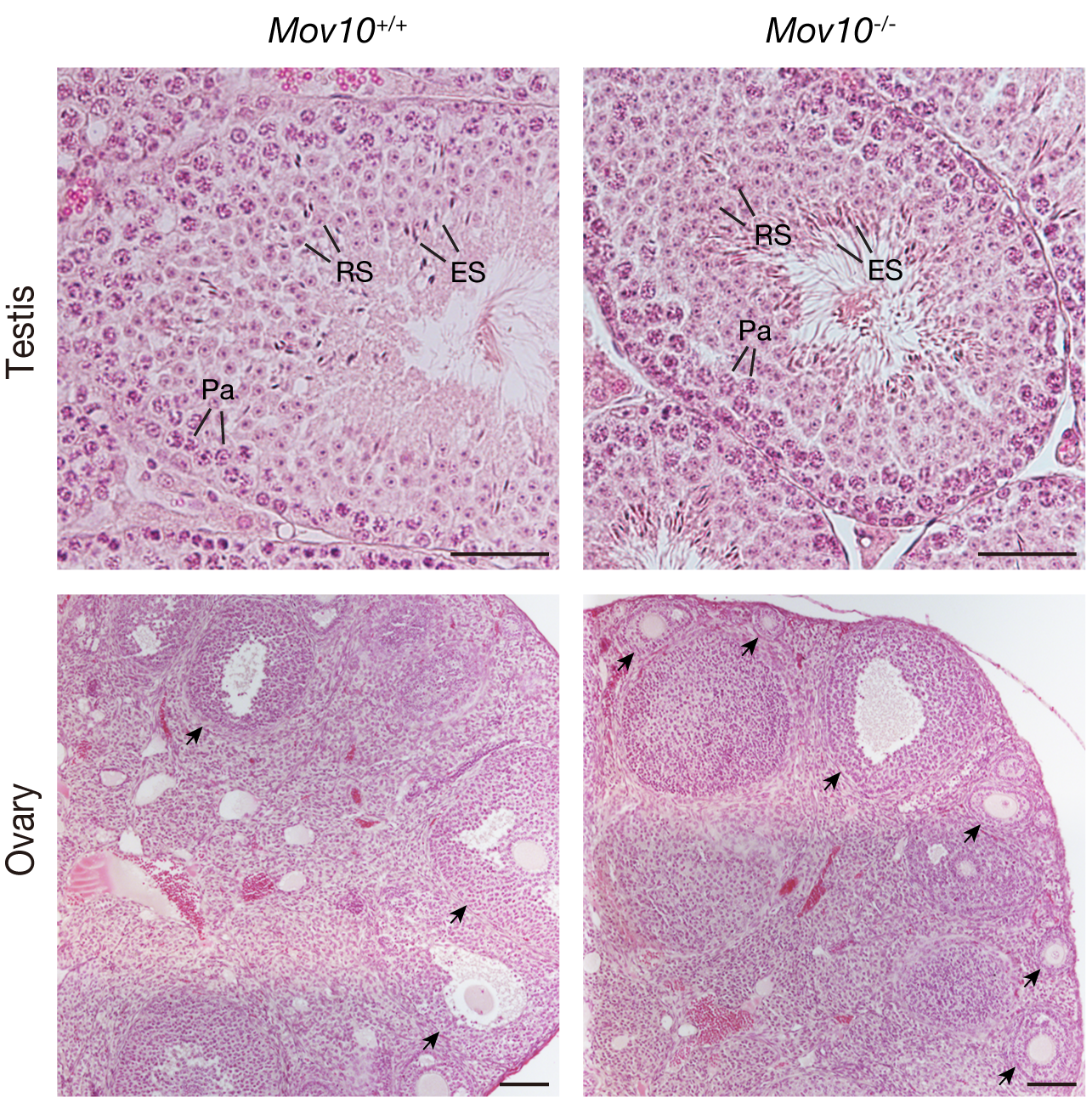

Supplement: S4 Fig — Arrows in the ovaries indicate follicles. Abbreviations: Pa, pachytene spermatocytes; RS, round spermatids; ES, elongated spermatids. Scale bars, 50 μm. (TIF) [file pgen.1010566.s004.tif]
