# Supplementary material for: The MOV10 RNA helicase is a dosage-dependent host restriction factor for LINE1 retrotransposition in mice
Source: PLoS Genet. 2023 May 1;19(5):e1010566. doi: 10.1371/journal.pgen.1010566 (PMC10174503; doi:10.1371/journal.pgen.1010566)
Supplement: S3 Table — (DOCX) [file pgen.1010566.s007.docx]

**S3 Table. Primary and secondary antibodies.**

| Antibody | Host | Producer | Cat. No/reference | Dilution | |
| --- | --- | --- | --- | --- | --- |
|  |  |  |  | WB | IF |
| MOV10 | Rabbit | Bethyl lab | A301-571A | 1:1000 | 1:300 |
| UPF1 | Rabbit | Bethyl lab | A301-902A | 1:1000 |  |
| ACTB | Mouse | Sigma | A5441 | 1:5000 |  |
| TEX19 | Rabbit | Custom-made | [67] | 1:500 |  |
| Histone H3 | Rabbit | Cell Signalling | 9715S | 1:2000 |  |
| SYCP3 | Rabbit | Preoteintech | 23024-I-AP | 1:1000 |  |
| Anti-Rabbit IgG Fluorescein | Goat | Vector Laboratories | FI-1000 |  | 1:200 |
| Anti-mouse IgG,  HRP-linked Antibody | Horse | Cell Signaling | 7076S | 1:5000 |  |
| Anti-rabbit IgG,  HRP-linked Antibody | Goat | Cell Signaling | 7074S | 1:5000 |  |
