# Supplementary material for: The MOV10 RNA helicase is a dosage-dependent host restriction factor for LINE1 retrotransposition in mice
Source: PLoS Genet. 2023 May 1;19(5):e1010566. doi: 10.1371/journal.pgen.1010566 (PMC10174503; doi:10.1371/journal.pgen.1010566)
Supplement: S4 Table — (DOCX) [file pgen.1010566.s008.docx]

**S4 Table.** **Primers used in MOV10 HITS-CLIP.**

| Primer name | Primer sequence |  | | |  |  |
| --- | --- | --- | --- | --- | --- | --- |
|  |  |  |  |  |  |  |
| RL3 (-P) | 5′-OH GUGUCAGUCACUUCCAGCGG 3′-Inverted dT | |  |  | |  |
| RL3 (+P) | 5′-p GUGUCAGUCACUUCCAGCGG 3′-Inverted dT | |  | |  | |
| RL5 | 5′-OH AGGGAGGACGAUGCGGNNNNG 3′-OH | |  | |  | |
| DP3 | 5′-CCGCTGGAAGTGACTGACAC-3′ | |  | |  | |
| DP5 | 5′-AGGGAGGACGATGCGG-3′ | |  | |  | |
| DSFP3 | 5′-CAAGCAGAAGACGGCATACGACCGCTGGAAGTGACTGACAC-3′ | |  | |  | |
| DSFP5-CGTA | 5′-AATGATACGGCGACCACCGAGATCTACACTCTTTCCCTACACG  ACGCTCTTCCGATCTCGTAAGGGAGGACGATGCGG-3′ | |  | |  | |
| DSFP5-GATC | 5′-AATGATACGGCGACCACCGAGATCTACACTCTTTCCCTACACG  ACGCTCTTCCGATCTGATCAGGGAGGACGATGCGG-3′ | |  | |  | |
| DSFP5-GTGC | 5′-AATGATACGGCGACCACCGAGATCTACACTCTTTCCCTACACG  ACGCTCTTCCGATCTGTGCAGGGAGGACGATGCGG-3′ | |  | |  | |
| DSFP5-CTAG | 5′-AATGATACGGCGACCACCGAGATCTACACTCTTTCCCTACACG  ACGCTCTTCCGATCTCTAGAGGGAGGACGATGCGG-3′ | |  | |  | |
