# Supplementary material for: The MOV10 RNA helicase is a dosage-dependent host restriction factor for LINE1 retrotransposition in mice
Source: PLoS Genet. 2023 May 1;19(5):e1010566. doi: 10.1371/journal.pgen.1010566 (PMC10174503; doi:10.1371/journal.pgen.1010566)
Supplement: S5 Table — (DOCX) [file pgen.1010566.s009.docx]

**S5 Table. RNA-seq datasets from postnatal day 21 (P21) mouse tissues used in Fig 2E.**

| **Tissue** | **Age** | **BioProject Accession** | **References** |
| --- | --- | --- | --- |
| Testis | P21 | PRJNA282462 | [1] |
| Testis | P21 | PRJNA298627 | [2] |
| Testis | P21 | PRJNA362514 | [3] |
| Testis | P21 | PRJNA138249 | [4] |
| Testis | P21 | PRJNA433934 | N/A |
| Kidney | P21 | PRJNA414151 | [5] |
| kidney | P21 | PRJNA285844 | [6] |
| Brainstem | P21 | PRJNA401713 | [7] |
| Bone | P21 | PRJNA490833 | [8] |
| Retina | P21 | PRJNA226093 | [9] |
| Retina | P21 | PRJNA269130 | [10] |
| Retina | P21 | PRJNA149389 | [11] |
| Retina | P21 | PRJNA363051 | [12] |
| Bone marrow | P21 | PRJNA523925 | N/A |
| Liver | P21 | PRJNA433590 | N/A |
| Cortex | P21 | PRJNA317986 | [13] |

**References**

1. Kistler WS, Baas D, Lemeille S, Paschaki M, Seguin-Estevez Q, Barras E, et al. RFX2 Is a Major Transcriptional Regulator of Spermiogenesis. PLoS Genet. 2015;11(7):e1005368. Epub 20150710. doi: 10.1371/journal.pgen.1005368. PubMed PMID: 26162102; PubMed Central PMCID: PMCPMC4498915.

2. Li W, Park JY, Zheng D, Hoque M, Yehia G, Tian B. Alternative cleavage and polyadenylation in spermatogenesis connects chromatin regulation with post-transcriptional control. BMC Biol. 2016;14:6. Epub 20160122. doi: 10.1186/s12915-016-0229-6. PubMed PMID: 26801249; PubMed Central PMCID: PMCPMC4724118.

3. Yang X, Yang Y, Sun BF, Chen YS, Xu JW, Lai WY, et al. 5-methylcytosine promotes mRNA export - NSUN2 as the methyltransferase and ALYREF as an m(5)C reader. Cell Res. 2017;27(5):606-25. Epub 20170418. doi: 10.1038/cr.2017.55. PubMed PMID: 28418038; PubMed Central PMCID: PMCPMC5594206.

4. Vourekas A, Zheng Q, Alexiou P, Maragkakis M, Kirino Y, Gregory BD, et al. Mili and Miwi target RNA repertoire reveals piRNA biogenesis and function of Miwi in spermiogenesis. Nature structural & molecular biology. 2012;19(8):773-81. doi: 10.1038/nsmb.2347; 10.1038/nsmb.2347.

5. Zhao J, Lupino K, Wilkins BJ, Qiu C, Liu J, Omura Y, et al. Genomic integration of ERRgamma-HNF1beta regulates renal bioenergetics and prevents chronic kidney disease. Proc Natl Acad Sci U S A. 2018;115(21):E4910-E9. Epub 20180507. doi: 10.1073/pnas.1804965115. PubMed PMID: 29735694; PubMed Central PMCID: PMCPMC6003475.

6. Lakhia R, Hajarnis S, Williams D, Aboudehen K, Yheskel M, Xing C, et al. MicroRNA-21 Aggravates Cyst Growth in a Model of Polycystic Kidney Disease. J Am Soc Nephrol. 2016;27(8):2319-30. Epub 20151217. doi: 10.1681/ASN.2015060634. PubMed PMID: 26677864; PubMed Central PMCID: PMCPMC4978047.

7. Lizen B, Moens C, Mouheiche J, Sacre T, Ahn MT, Jeannotte L, et al. Conditional Loss of Hoxa5 Function Early after Birth Impacts on Expression of Genes with Synaptic Function. Front Mol Neurosci. 2017;10:369. Epub 20171115. doi: 10.3389/fnmol.2017.00369. PubMed PMID: 29187810; PubMed Central PMCID: PMCPMC5695161.

8. Quarto N, Shailendra S, Meyer NP, Menon S, Renda A, Longaker MT. Twist1-Haploinsufficiency Selectively Enhances the Osteoskeletal Capacity of Mesoderm-Derived Parietal Bone Through Downregulation of Fgf23. Front Physiol. 2018;9:1426. Epub 20181015. doi: 10.3389/fphys.2018.01426. PubMed PMID: 30374308; PubMed Central PMCID: PMCPMC6196243.

9. Roger JE, Hiriyanna A, Gotoh N, Hao H, Cheng DF, Ratnapriya R, et al. OTX2 loss causes rod differentiation defect in CRX-associated congenital blindness. J Clin Invest. 2014;124(2):631-43. Epub 20140102. doi: 10.1172/JCI72722. PubMed PMID: 24382353; PubMed Central PMCID: PMCPMC3904630.

10. Kevany BM, Zhang N, Jastrzebska B, Palczewski K. Animals deficient in C2Orf71, an autosomal recessive retinitis pigmentosa-associated locus, develop severe early-onset retinal degeneration. Hum Mol Genet. 2015;24(9):2627-40. Epub 20150123. doi: 10.1093/hmg/ddv025. PubMed PMID: 25616964; PubMed Central PMCID: PMCPMC4383867.

11. Brooks MJ, Rajasimha HK, Roger JE, Swaroop A. Next-generation sequencing facilitates quantitative analysis of wild-type and Nrl(-/-) retinal transcriptomes. Mol Vis. 2011;17:3034-54. Epub 20111123. PubMed PMID: 22162623; PubMed Central PMCID: PMCPMC3233386.

12. Campla CK, Breit H, Dong L, Gumerson JD, Roger JE, Swaroop A. Pias3 is necessary for dorso-ventral patterning and visual response of retinal cones but is not required for rod photoreceptor differentiation. Biol Open. 2017;6(6):881-90. Epub 20170615. doi: 10.1242/bio.024679. PubMed PMID: 28495965; PubMed Central PMCID: PMCPMC5483026.

13. Li Q, Guo S, Jiang X, Bryk J, Naumann R, Enard W, et al. Mice carrying a human GLUD2 gene recapitulate aspects of human transcriptome and metabolome development. Proc Natl Acad Sci U S A. 2016;113(19):5358-63. Epub 20160426. doi: 10.1073/pnas.1519261113. PubMed PMID: 27118840; PubMed Central PMCID: PMCPMC4868425.
