# Supplementary material for: The MOV10 RNA helicase is a dosage-dependent host restriction factor for LINE1 retrotransposition in mice
Source: PLoS Genet. 2023 May 1;19(5):e1010566. doi: 10.1371/journal.pgen.1010566 (PMC10174503; doi:10.1371/journal.pgen.1010566)
Supplement: Attachment — Submitted filename: ResponseToReviewsRevision2.pdf [file pgen.1010566.s012.pdf]

Alex Bortvin, M.D., Ph.D.
Guest Editor
PLOS Genetics

Gregory Barsh
Editor-in-Chief
PLOS Genetics

Re: PGENETICS-D-22-01422R1

April 3, 2023

Dear Drs. Bortvin and Barsh,

Thank you for the review of our revised manuscript. We have addressed the remaining minor critique of Reviewer 3.

Reviewer #1: I commend the authors for the revision. I find the revised version to my satisfaction. It will contribute to our evolving understanding of the protein.

Reviewer #2: The authors have addressed all my questions.

Reviewer #3: The manuscript is greatly improved and I only have one point of concern regarding figures 2C/2D. It would be useful to include in the materials how this read density was normalized. For example, how does the read density of L1_MM shown in figure 2D compare to the other transcripts displayed in figure 2C? It is hard to compare without units on the y-axis. How would the data of figure 2D look if superimposed on figure 2C? Also, the text says that MOV10 CLIP-seq density is enriching in the L1 3' UTR, but the pattern is strikingly different between figures 2C and 2D, with most transcripts having a huge bump is read density throughout the 3'UTR compared to coding regions. The L1 actually has very similar levels of read density of the 3' UTR and coding region, with the exception of the terminus of the 3'UTR (which may be the polyA tail?). Does figure 2D take into account that the L1 3' end is more abundant in the genome and thus in total RNA?

I do not need to see the manuscript again assuming this concern is adequately answered.

<u>Response</u>: We appreciated Reviewer 3's detailed suggestions. We have added read density normalization to the Materials and Methods: "The read density of each CLIP-binding transcript was normalized to reads per million reads (RPM) with the formula: RPM = (number of reads mapped to transcripts/total number of mapped reads) × 1,000,000" (Page 15, lines 446-448). We have also added units on the Y-axis in Fig 2C and Fig 2D as shown on the right. The read density of L1_MM (Fig 2D) is 10 times higher than the read density of other protein-coding transcripts (Fig 2C). Indeed, the CLIP-seq read density increased throughout the 3'UTR in other protein coding transcripts (Fig 2C), however, L1_MM has similar levels in CDS and 3'UTR with only a sharp increase toward the end of L1_MM 3'UTR (Fig 2D).

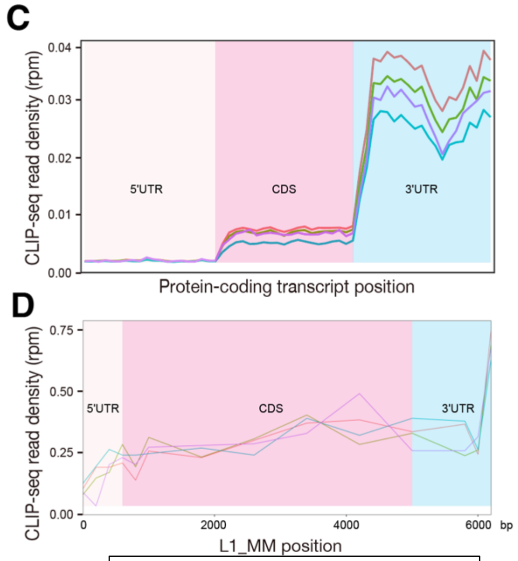

Fig 2C and Fig 2D

We checked that the spike of CLIP-seq reads in L1_MM 3'UTR terminus do not correspond to poly(A) tail. Then we analyzed the read density of L1_MM in postnatal day 21 testis (RNA-seq data) (shown in Fig X; for review purpose only). The L1_MM transcript in testis is highly abundant (40 RPM). Superimposed with the RNA-seq profile, the CLIP-seq profile of L1_MM transcript is nearly a flat line. The CLIP-seq read bump close to the end of L1_MM 3'UTR corresponded to a sharp decrease in the RNA-seq read density (indicated by an arrow, Fig X). We couldn't explain this spike to the 3'UTR end in L1_MM CLIP-seq reads. We didn't observe this spike in other L1 families. We cannot rule out the possibility that it might be a CLIP-seq artifact. Given these uncertainties and taking into account of Reviewer 3's concern, we decided to delete Fig 2D (CLIP-seq read density map of L1_MM). Accordingly, we have also deleted the following statement from the Results: "MOV10 CLIP tags were also enriched in 3' UTRs of the L1_mm family but not other L1 families". The deletion of Fig 2D does not affect the conclusions of this study.

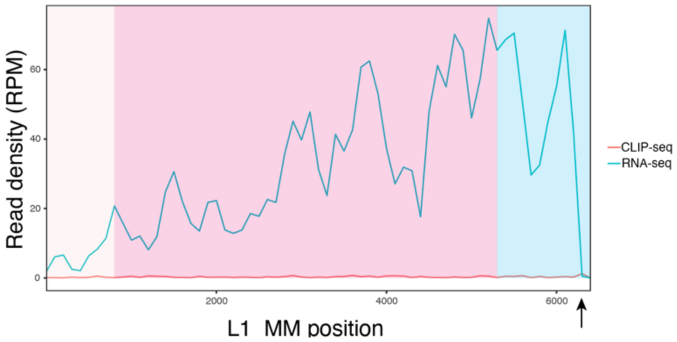

Fig X: Distribution of MOV10 CLIP tags (read line) and RNA-seq reads (light blue) from postnatal day 21 mouse testes along the L1-MM retrotransposon.

I hope that the revised manuscript is now suitable for publication.

Sincerely yours,

Jeremy Wang
