# Supplementary material for: The MOV10 RNA helicase is a dosage-dependent host restriction factor for LINE1 retrotransposition in mice
Source: PLoS Genet. 2023 May 1;19(5):e1010566. doi: 10.1371/journal.pgen.1010566 (PMC10174503; doi:10.1371/journal.pgen.1010566)
Supplement: Attachment — Submitted filename: ResponseToReviews.docx [file pgen.1010566.s011.docx]

Alex Bortvin, M.D., Ph.D.

Guest Editor

PLOS Genetics

Gregory Barsh

Editor-in-Chief

PLOS Genetics

Re: PGENETICS-D-22-01422

March 6, 2023

Dear Drs. Bortvin and Barsh,

Thank you for the opportunity to revise our manuscript! We’d like to thank all the reviewers for their comments. We have addressed all the concerns with new data, new analyses, and text revisions.

Please note that Zhenlong Kang has been added as a co-author, as he contributed to the revision. All authors have agreed with this addition.

We have added the following new figures/Tables:

Fig 1A: GAPDH WB loading control was added.

Fig 2D and 2H: MOV10 CLIP tags in L1_mm (Fig 2D) and GO analysis of MOV10-bound transcripts (Fig 2H).

Fig 3D: GO analysis of DE genes.

Fig 4C: a plot was added.

S4 Fig: Histology of testes and ovaries from 8-week-old wild type and *Mov10*^-/-^ mice.

S5 Table. RNA-seq datasets from postnatal day 21 (P21) mouse tissues.

A marked up copy of the manuscript has also been uploaded.

Below is our point-by-point response to the reviewers’ comments.

**Reviewer #1:**
The authors investigate the role of an RNA helicase MOV10 in mice and describe its role in retrotransposon repression. The authors demonstrate a role that is distinctly different from that reported previously by the authors for its paralog MOV10L1. Loss of Mov10 causes low-penetrant lethality, but those who make it beyond the embryonic stages appear normal. They identify MOV10-bound transcripts to show that the protein binds the 3’UTR of several mRNAs and a select set of L1 retrotransposons, and these L1 elements are upregulated in the KO germline. Using a L1 transposition reporter, they identify increased transposition events in the KO mouse tissues, that is also seen at reduced levels in Mov10 HET animals, indicating a dosage effect. These transposition events result in increased impact on gene expression changes over successive generations, perhaps due to accumulation of mutations. Taken together, this nicely executed study demonstrates a role for MOV10 in retrotransposon control using the mouse model and quantifies it using a L1 transposition reporter. A role for regulation of non-germline transcripts (mainly soma-expressed) is also proposed. I support its publication after the minor concerns are addressed.

Minor.
1. The role of UPF1 in L1 inhibition by MOV10 should be clearly discussed. UPF1 is also shown to bind to mRNA 3’UTRs (Hurt et al., 2013; Fiorini et al., 2015) that may remodel protein complexes on the RNA. Since MOV10 and UPF1 can bind RNA independently, is there any overlap of RNAs bound by the two proteins? I don’t know if dataset of CLIPseq of UPF1 in mouse testis is available? Please compare and discuss, if such a dataset is available.

**Response**: There are no dataset of UPF1 CLIP-seq in mouse testis. Therefore, we compared with the dataset of UPF1 CLIP-seq in mouse ES cells (Hurt et al., 2013). Hurt *et al* reported 210 high-confident UPF1-bound RNA targets. 41 of these UPF1 targets (20%) are also present in our MOV10 CLIP targets (2305 transcripts; Fold enrichment>10). We have included this comparison in the Results. We have also expanded the discussion on the role of UPF1 as shown by these two studies.

2. Line 154: The statement should refer to the figure where this data is shown, if it exists? I assume it’s unpublished data or part of Figure S3C?

**Response**: We have now included these data (histology of *Mov10*^-/-^ testis and ovary) as a new supplementary figure – Fig S4.

3. Line 182-193: It is not clear if the statements convey the intended meaning. This is what I understand, MOV10 binds RNAs which are degraded. In the Mov10 mutant testis, these RNAs are upregulated (Fig 3D). Please check.

**Response**: By analyzing the entire MOV10 bound population, we did not find a correlation between MOV10 binding strength and differential expression changes. We have revised this section but refrained from drawing a correlation.

4. Line 198: Again, there is a contradiction in the two statements on L1 and SINE elements being regulated by MOV10. Please check.

**Response**: Reviewer 3 also raised this concern. We have deleted these analyses.

5. In the results section, please clearly state where the conditional KO is used vs the clean KO.

**Response**: We have now clearly stated these. The clean KO was used in all figures except Fig. S3C where conditional knockout was used.

**Reviewer #2:**

In this manuscript, the authors used Mov10 knockout and conditional knockout mouse models and L1 retrotransposition reporter mice to evaluate a role for Mov10 in inhibiting L1 retrotransposition in vivo. They reveal that unlike Mov10l1, an Mov10 family member that regulates L1 through the piRNA pathway, Mov10 suppresses L1 transposition in various mouse tissues. This is a significant advance in understanding the distinctive mechanisms for the Mov10 family RNA helicases in L1 regulation.

Comments:

It would be helpful to show GFP protein expression data in Mov10-/- L1tg/tg tissues such as GFP immunofluorescence, and/or LINE1 immunofluorescence in Mov10-/- L1 tissue. This will further strengthen the conclusion.

**Response**: We have examined the L1-retrotransposition-positive *Mov10*^-/-^ L1^tg/tg^ kidney tissues for green fluorescence of GFP and by immunostaining of tissue sections with anti-GFP antibodies. We failed to observe expression of GFP. We contacted Dr. Wenfeng An who originally produced the L1^tg^ mice. Dr. An’s lab showed that L1 retrotransposition was increased in the *Mov10l1*-deficient (piRNA-deficient) mice using this L1^tg^ reporter (Newkirk et al., PNAS, 2017; Ref 55). Dr. An’s lab did not observe GFP signals either. There are several possibilities for the failure in the GFP expression. First, it is possible that the transcriptional and translational elements for GFP in the L1^tg^ do not function efficiently in tissues (Fig. 4A). Second, new L1 insertions are mostly truncated and thus some control elements might be missing. Third, new L1 insertions might have landed in transcriptionally non-permissive regions.

Line 284-301: the authors indicate that MOV10 is a “potent” inhibitor of L1 retrotransposition in mouse (in vivo) and MOV10 regulates L1 retrotransposition through several mechanisms. How to define “potent”, this should be backed by data.

**Response**: Since MOV10 is the only known inhibitor of L1 retrotransposition in mouse (in vivo), we cannot compare the relative potency. Therefore, we have removed the description “potent”.

Line 260: “G2 Mov10-/- mice exhibited significant reductions in testis weight, sperm count, and the percentage of abnormal seminiferous tubules.” Should be an increase of percentage of abnormal seminiferous tubules.

**Response**: Corrected.

Is reproductive fitness defect described in Fig. 5 the result of misregulation of L1 retrotransposition? How would the authors explain L1 unrelated function of Mov10 in germ cell regulation?

**Response**: We are uncertain about the cause of reproductive defects in *Mov10* KO mice. They are more likely to be caused by loss of L1-unrelated functions than misregulation of L1 retrotransposition or by a combination of both. We have revised the discussion paragraph (2^nd^ to last) and discussed these possibilities.

The model in Fig. 6: can the author exclude any overlapping function between Mov10 and Mov10l1 in piRNA biogenesis or in L1 retrotransposition?

**Response**: The functions of MOV10 and MOV10L1 appear to be largely separate. MOV10L1 is germ cell-specific, but MOV10 is ubiquitously expressed. The inhibition of L1 retrotransposition in somatic tissues is specific to MOV10. MOV10L1 is essential for piRNA biogenesis in germ cells. MOV10 is not essential for piRNA biogenesis as the *Mov10* KO mice lacked meiotic arrest, a hallmark of piRNA pathway mutants. However, although less likely, we cannot rule out the possibility that MOV10 might play a minor and non-essential role in piRNA biogenesis in germ cells. In addition, we cannot rule out the possibility that MOV10L1 might play a role in L1 retrotransposition in germ cells in addition to piRNA biogenesis. We have added the following to Discussion (the end of 3^rd^ paragraph in Discussion): “However, we cannot rule out a possible overlap of function of MOV10 and MOV10L1 in piRNA biogenesis and L1 retrotransposition in germ cells.”

**Reviewer #3:**

The paper would be of interest to the fields of mobile elements, virology, and development. Based on the title and abstract, the main conclusions the authors want to put forth are that MOV10 KO leads to L1 up regulation in testes, and subsequently L1 retrotransposition. I think this is a good first effort however the depth of analysis, conclusions, and data presentation needs to be strengthened. There does seem to be a clear trend that there is more retrotransposition in MOV10-/- mice, but given that MOV10 seems to bind to and affect so many transcripts, this modest retrotransposition increase could very well be an indirect effect. Other descriptions of the MOV10 phenotype are given (incomplete embryonic lethality, reduced reproductive fitness over successive generations), but it is unclear whether these are related to the L1 phenotype. Retrotransposition is occurring at quite low frequencies in wt, so the slight bump in frequency may not have any obvious biological consequence in the experiments shown.

Points of concern:
1) There is already a published mouse KO of MOV10 (Skariah et al 2017), but the authors inexplicably do not mention this until the end of their manuscript, halfway through the discussion. Plausible explanations for the differences in embryonic lethality are given, such as potentially different background, but then the authors claim that Skariah et al do not report the genetic background of their mouse which is incorrect (the background is C57BL/ 6 as described in additional file 3). Although the Skariah et al paper has some dubious conclusions (e.g. the “doubling of genomic L1 in MOV10 heterozygotes” is highly suspect), the existence of this literature should be acknowledged and differences or problems with the paper mentioned upfront. The focus of Skariah et al was different from this manuscript so it should not negatively affect this manuscript.

**Response**: We appreciate this correction and apologize for missing the genetic background for the *Mov10* mutant mice in the Skariah study. One possible explanation for the difference in penetrance of embryonic lethality could be the difference in genetic backgrounds (hybrid versus inbred). We have now described the Skariah study in Introduction in addition to Discussion.

2) Line 115 why is b-actin used as a loading control when it is barely expressed and heart/ skeletal muscle? How can you make conclusions about MOV10 expression in heart/muscle based on this figure?

**Response**: We now used GAPDH as a loading control and repeated WB with anti-GAPDH antibody. GAPDH is expressed in both heart and skeletal muscle. MOV10 expression pattern remains the same in the repeat. We have added GAPDH WB to Fig. 1A as an additional loading control.

3) Figure S1. There appear to be many bands specific to the MOV10 IP. What was the rationale for only choosing 4 bands to identify? What are the other bands besides UPF1? Why highlight other bands that were identified without saying what they are?

**Response**: We chose the strongest bands in MOV10 IP for mass spec. We chose a total of four bands. Two bands corresponded to MOV10 and UPF1 respectively. The other two weaker bands did not reveal proteins that made sense. Therefore, we did not sequence additional weaker bands. We have revised Figure S1. Only two relevant bands are now highlighted.

4) Figure 2 - Although the authors describe the general patterns on MOV10 RNA binding (preferentially binds to 3’UTR, binds to low abundance RNAs), I’m puzzled as to why they did not go into more depth on the names of the RNAs that were bound. Do they fall into any particular functional categories? Can they explain the MOV10 phenotype? Are retroelement sequences overrepresented in the MOV10 strong binders? It seems like a lot of work to produce this data, only to gloss over the details.

**Response**: We have performed GO analysis of MOV10 highly bound transcripts (enrichment fold > 10) and presented this analysis in a new panel – Fig. 2H. The top enriched GO categories are regulation of neurogenesis, ameboidal-type cell migration, respiratory system development, muscle tissue development, and etc. This suggests that MOV10 binds to somatically expressed transcripts in testes. All the MOV10 target RNAs from the CLIP-seq and GO results are shown in Table S1. The finding that MOV10 has so many somatic target transcripts might provide an explanation for the lethality in *Mov10* KO embryos. However, the penetrance of embryonic lethality is incomplete and the viable knockout mice were fertile. Given the complexity of the KO phenotypes, it doesn’t seem possible to correlate the CLIP-seq target with the phenotypes. We have also analyzed retrotransposons. We find that MOV10 CLIP tags were also enriched in 3’ UTRs of the L1_mm family but not other L1 families, which was presented in a new panel – Fig 2D.

5) I am confused by the interpretation of Figure 3. If the authors are proposing that MOV10 binds to and down regulates RNAs, why does the knockout of MOV10 lead to massive down regulation of the transcriptome? Figure 2D seems to show many, many RNAs that are low expression, strong MOV10 binding, but then knockout of MOV10 only leads to 60 RNAs being unregulated while >900 are down regulated? Figure 3D picks 20 transcripts that are strongly bound to MOV10 and have increased expression in the MOV10 KO, but this seems to be cherry picking. Aren’t these a small minority of the MOV10 bound RNAs? What if the authors analyzed the entire MOV10 bound population?

**Response**: Indeed, knockout of MOV10 leads to downregulation of more than 900 genes but upregulation of only 60 genes in testes. This result does not support that MOV10 binding leads to transcript destabilization. We have deleted the panel (previously Fig 3D) that showed the analysis of 20 upregulated transcripts that are CLIP targets of MOV10, since the number of upregulated DE genes is much lower than the number of DOWN DE genes. We also analyzed the entire MOV10 bound population and the entire set of DE genes but failed to detect a correlation between binding strength and the differential expression changes. Therefore, we have refrained from drawing a correlation between MOV10 binding and the transcript abundance. In addition, GO analysis revealed that the down-regulated genes were highly enriched in several reproductive processes such as spermatid differentiation, fertilization, and sperm motility (Fig 3D), whereas the up-regulated genes were moderately enriched in one GO term: negative regulation of viral process (the adjusted *p* value < 0.003; S2 Table). We have added the GO results to S2 Table and added the GO analysis of downregulated genes in a new panel – Fig 3D.

6) The analysis of L1 transcription is problematic. There are many fragments of L1 in the genome that are incorporated into the transcript of other genes. Some of the most abundant transcripts containing L1 are not bona fide L1 transcription, but fragments of L1 in the 3’ UTR of an mRNA. The analysis pipeline used to measure L1 family transcripts does not take this locus specific information into account and thus we do not know if these increases represent an increase of authentic L1 transcription. I don’t believe any solid conclusions can be made about L1 expression from this analysis, and the same could be possibly be said about the other elements.

**Response**: We agree. We have deleted the expression analysis of individual repeat families (L1 and SINE) from Figure 3 and the results section.

7) Figure 4 - there seems to be redundancy in the subfigures. Why not combine the 8 week KO samples from figure 4C and 4D? Are they essentially the same? It is hard to make conclusions about which tissues are higher or lower, as the percentages for same tissues and genotype are very different in some cases (e.g. adult KO lung is <10% in figure 4C but presumably the same experiment adult KO lung is 30% in figure 4D).

**Response**: The adult KO mice in Figure 4C were different from the adult KO mice in Figure 4D. The mice of three genotypes in Figure 4D were littermates. The percentage in some tissues such as lung has a larger range and is more variable, possibly due to the low frequency of L1 insertions. However, the percentage in three tissues (liver, kidney, and spleen) was consistently high in KO mice. To better present the data in Figure 4C, we have added a dot plot.

Minor comments:
- Where appropriate, please cite primary literature. For example, line 75 Dewannieux and Heidmann (2003 and 2005) are more appropriate citations than references 12 and 13.
- Line 81 Carmell et all (2006) and some others should be added as primary references.
- Line 97 Moldavan and Moran (2015) is another proteomic study that should be cited.

**Response**: We have cited all these references and other primary references.

- line 730 (Figure 2D legend) what is 3 weeks and what is adult in this subfigure?

**Response**: Only data from 3-week-old mice were used for this analysis. We have deleted “adult” from the legend. We apologize for this error. We have listed all the tissues, BioProject Accession Number, and references in a new supplementary table (S5 Table).

- Lines 241-247. The experiment does not test germline transmission, so why even start this paragraph stating that you were trying to test germline transmission? I would just state the results. If this data (figure 4F) was broken down into the different pup genotypes, there might also be some interesting information there.

**Response**: We revised accordingly by just describing the results.

I hope that the revised manuscript is suitable for publication.

Sincerely yours,

Jeremy Wang
